# Improving wellbeing and reducing future world population

**DOI:** 10.1371/journal.pone.0202851

**Published:** 2018-09-12

**Authors:** William W. Murdoch, Fang-I Chu, Allan Stewart-Oaten, Mark Q. Wilber

**Affiliations:** 1 Department of Ecology, Evolution and Marine Biology, University of California Santa Barbara, Santa Barbara, CA, United States of America; 2 Department of Statistics and Applied Probability, University of California Santa Barbara, Santa Barbara, CA, United States of America; University of Indianapolis, UNITED STATES

## Abstract

Almost 80% of the 4 billion projected increase in world population by 2100 comes from 37 Mid-African Countries (MACs), caused mostly by slow declines in Total Fertility Rate (TFR). Historically, TFR has declined in response to increases in wellbeing associated with economic development. We show that, when Infant Survival Rate (ISR, a proxy for wellbeing) has increased, MAC fertility has declined at the same rate, in relation to ISR, as it did in 61 comparable Other Developing Countries (ODCs) whose average fertility is close to replacement level. If MAC ISR were to increase at the historic rate of these ODCs, and TFR declined correspondingly, then the projected world population in 2100 would be decreasing and 1.1 billion lower than currently projected. Such rates of ISR increase, and TFR decrease, are quite feasible and have occurred in comparable ODCs. Increased efforts to improve the wellbeing of poor MAC populations are key.

## Introduction

The UN’s median projected world population in the year 2100 is more than 11 billion and still increasing [1–3]. Just over 3 billion people, or 78.5% of the 3.86 billion projected world population increase from 2015 to 2100, come from the world’s poorest region: a band of 37 high-fertility Mid-African Countries (MACs), which include all African countries with at least 1 million people in 2000, except for the five northern and five southern low-fertility nations. All but Sudan are sub-Saharan. The median UN-projected 2100 MAC population is over 3.97 billion.

The great majority of sub-Saharan (and hence MAC) projected population increase comes from high and slowly declining fertility [4]. Sub-Saharan fertility decline started about 20 years later than that of other developing nations [5] and, once begun, is estimated to have been one-fourth as fast as in Asia and Latin America at the equivalent demographic stage [6].

Historically, fertility has declined in response to increases in wellbeing associated with economic development. We show that, when Infant Survival Rate (ISR, a proxy for wellbeing) has increased, MAC fertility has declined at the same rate, in relation to ISR, as it did in 61 comparable Other Developing Countries (ODCs) whose average fertility is now close to replacement level. We show that if MAC ISR were to increase at the historic rate of these ODCs, and fertility declined correspondingly, then the projected world population in 2100 would be decreasing and 1.1 billion lower than currently projected. Such rates of ISR increase, and fertility decrease, are quite feasible and have occurred in ODCs in conditions comparable to MACs in the present day. Increased efforts to improve the wellbeing of poor MAC populations are key.

## Approach and results

### Middle-African (MACs) and Other Developing Countries (ODCs)

**Fig 1:**
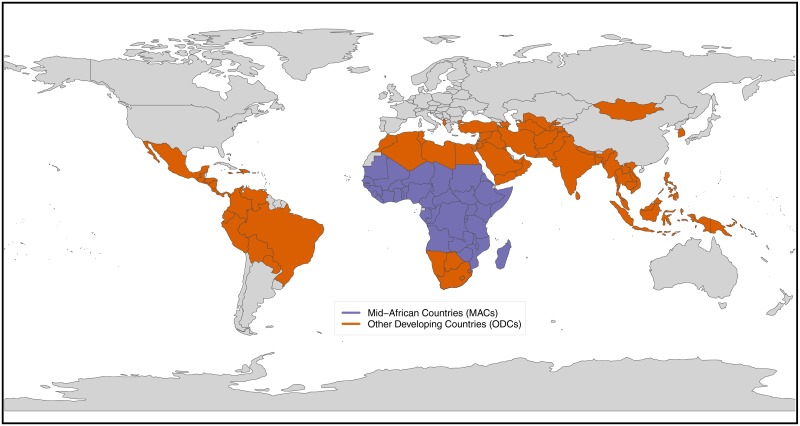
Nations in two comparable groups. **Mid-African Countries (MACs)**: Eastern Africa: Burundi, Eritrea, Ethiopia, Kenya, Madagascar, Malawi, Mozambique, Rwanda, Somalia, S. Sudan, Uganda, U.R. Tanzania, Zambia, Zimbabwe; Middle Africa: Angola, Cameroon, Central African Republic, Chad, Congo, D.R. Congo, Gabon; North Africa: Sudan; Western Africa: Benin, Burkina Faso, Cote d’Ivoire, Gambia, Ghana, Guinea, Guinea Bissau, Liberia, Mali, Mauritania, Niger, Nigeria, Senegal, Sierra Leone, Togo. **Other Developing Countries (ODCs)**: Northern Africa: Algeria, Egypt, Libya, Morocco, Tunisia; Southern Africa: Botswana, Lesotho, Namibia, South Africa, Swaziland; Eastern Asia: R. Korea, Mongolia; Central Asia: Tajikistan, Turkmenistan, Uzbekistan; Southern Asia: Afghanistan, Bangladesh, India, Iran, Nepal, Pakistan, Sri Lanka; S.E. Asia: Cambodia, Indonesia, Lao P.D.R., Malaysia, Myanmar, Philippines, Singapore, Thailand, Viet Nam; Western Asia: Azerbaijan, Iraq, Jordan, Kuwait, Oman, Saudi Arabia, State of Palestine, Syria, Turkey, U.A. Emirates, Yemen; Southern Europe: Albania; Caribbean: Dominican Republic, Haiti, Jamaica; Central America: Costa Rica, El Salvador, Guatemala, Honduras, Mexico, Nicaragua, Panama; South America: Bolivia, Brazil, Colombia, Ecuador, Paraguay, Peru, Venezuela; Melanesia: Papua New Guinea. The base map is from Natural Earth and is in the public domain under a Creative Commons license.

We compare MACs with the demographic history of 61 Other Developing Countries (ODCs) which had high fertility (Total Fertility Rate, TFR = 6 or greater in almost all cases) in 1950-55 (the first period for which UN world data are available), and which experienced all or almost all of their fertility decline thereafter (S1 Text). We excluded China from the ODCs because of its unique 1-child policy. As in the MACs, all ODCs had more than 1 million people in 2000. The ODCs represent all major geographical regions (Fig 1).

**Fig 2:**
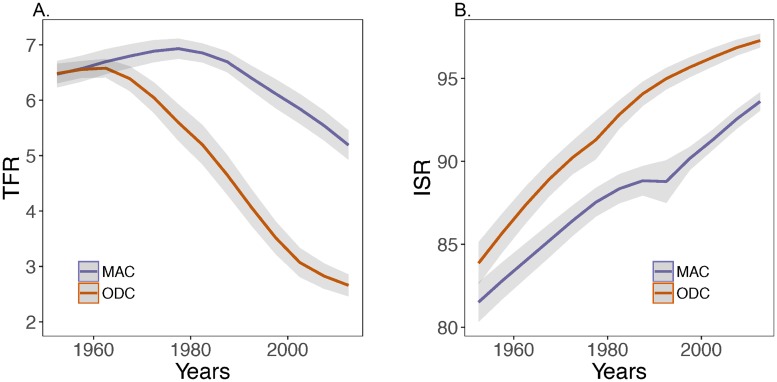
**A**. Mean Total Fertility Rate (TFR) and **B**. mean Infant Survival Rate (ISR) with 95% confidence limits for Mid-African Countries (MAC) and Other Developing Countries (ODC), over time since 1950-55. Data from [3].

The average TFR for MACs in 2015 was 5.19, more than twice both the ultimate replacement rate and the overall world average (2.51) [3], and almost twice the ODC average (2.66). As seen in the sub-Saharan comparisons discussed above [6], MAC fertility decline has been later and slower than in the ODCs (Fig 2A).

The different MAC and ODC fertility trajectories suggest there may be some major causal differences [6]. [7] shows that fertility decline in sub-Saharan Africa began at an unusually low level of economic and social development. He also shows that the higher sub-Saharan African fertility since 1960 is correlated with a higher desired family size (though [8] note that, recently, desired family size has been declining in the region). However, we suggest that the main drivers of fertility decline operate in the same way in ODCs and MACs: that decrease in fertility is a response to the level and rate of change of the population’s general wellbeing.

### Wellbeing and fertility

A vast body of evidence shows that desired family size is determined rationally and, beginning in Europe in the late nineteenth century, has declined largely in response to increased parental socio-economic wellbeing, including associated changes in the costs and benefits of children [7–11]. A diffusion effect, in which fertility decline spreads within a culture may also have operated in some situations [7]. Note that at the lowest levels of development, fertility often first increases with improved wellbeing (e.g. MACs in Fig 2A), but thereafter has typically declined steadily.

**Fig 3:**
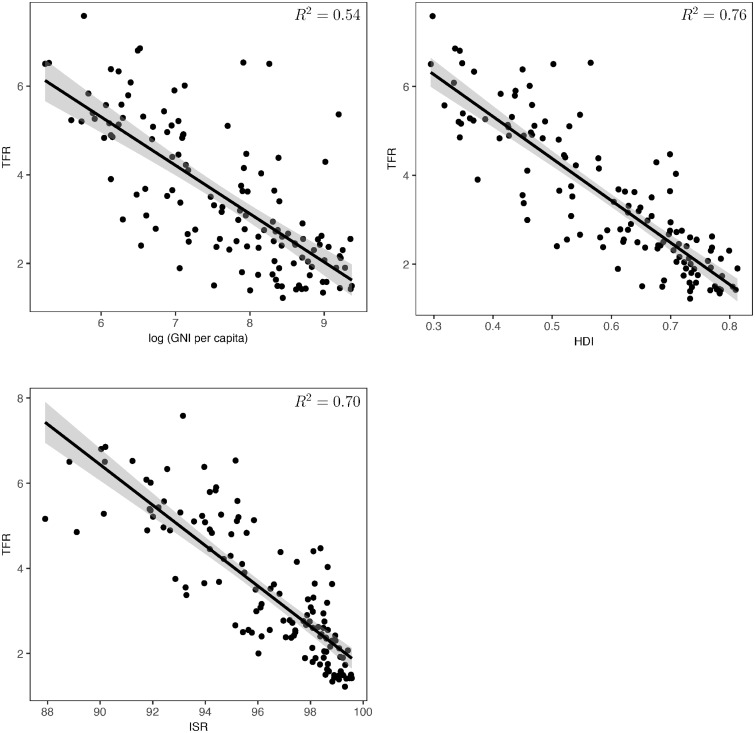
**A**. Linear regressions (and 95% confidence bands) of TFR vs log(GNI per capita) (referred to as “per capita income” in the main text) for all developing countries in 2010 as defined by the World Bank [13]. Income data from World Bank [14] and TFR data from United Nations [3]. **B**. Linear regression (and 95% confidence bands) of TFR on Human Development Index (HDI) in 2010, for all countries in Fig 3 for which an HDI value is available [12]. **C**. Linear regression (and 95% confidence bands) of TFR on ISR in 2010. TFR and ISR data from United Nations [3].

We next illustrate briefly the relationship between fertility and wellbeing. First, about half (54%) of the variation in TFR among developing countries at one point in time is explained by variation in log(per capita income) (Fig 3A). But per capita income misses a major aspect of *general* wellbeing, namely how widely income and the benefits of development, such as improved health and education, are spread across the population. The UN Human Development Index [12], HDI, which combines per capita income with scores representing levels of health and education explains about 75% of the variation in TFR (Fig 3B).

Second, [7] conducted a wide-ranging analysis of development and fertility patterns in sub-Saharan Africa. He suggests that fertility has broadly responded to development in sub-Saharan Africa as in 52 other developing countries, with the provisos noted above that sub-Saharan African’s fertility began to decline at a lower level of development, but usually remained higher at each particular level.

Finally, in 25 sub-Saharan countries with DHS (Demographic and Health Surveys) data from multiple time periods, fertility fell significantly faster between consecutive surveys in countries that experienced a greater increase in female education and in those that experienced a greater reduction in infant and child mortality [5].

### Infant survival rate and general wellbeing

We use Infant Survival Rate, ISR = the percent of infants who survive to their first birthday, as our proxy for wellbeing (see S2 Text for other possible measures). It is equivalent to the Infant Mortality Rate (ISR = 100—IMR/10) used by the UN (see [3]). ISR is likely to indicate not only the health of infants, but also other components of wellbeing such as general health, access to medical care, other services, information, and other goods or opportunities. There is more historical data for ISR than for HDI (described above) and it explains statistically about 70% of the variance in TFR (Fig 3C). It is a particularly good indicator of improvement in *general* population wellbeing, as we explain next.

Data collected during the Demographic and Health Surveys (DHS) illustrate the relationship between national ISR values and general wellbeing in the population. The surveys measure ISR in families that are also classified into five equal-sized relative “wealth” classes. Wealth, a proxy for income, is measured by an index of household conditions and goods [15].

**Fig 4:**
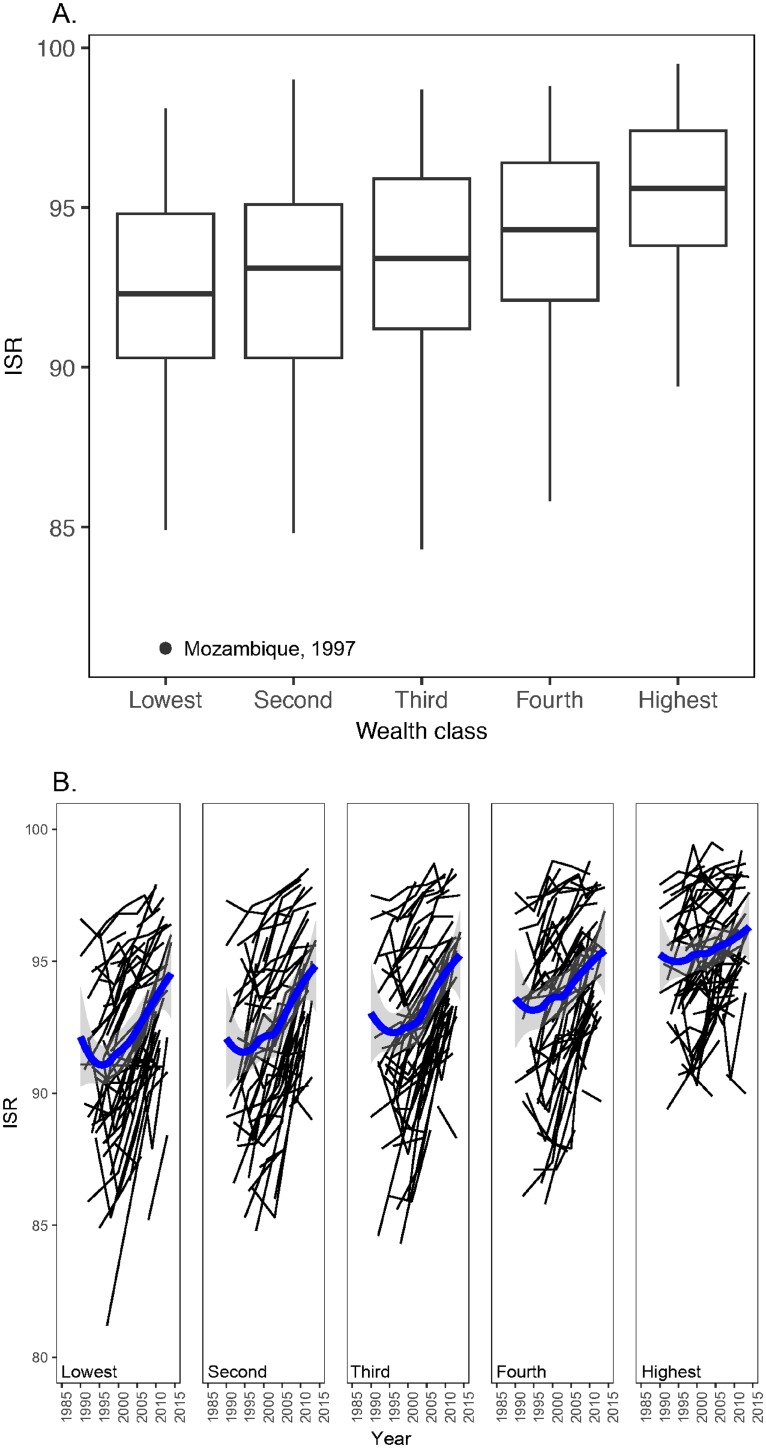
**A**. The boxplot summarizes the relation between ISR and relative wealth. For each of 72 developing countries, we obtained ISR values from 1990 to 2014 for the 5 wealth classes. For each wealth class, we combined the values across countries for the box plots (the sequence of medians from Lowest to Highest is: 92.3, 93.1, 93.4, 94.3 and 95.6). Using the medians for each country’s wealth classes, a Friedman test that accounts for the within country correlation showed a significant difference among wealth classes (*χ*^2^ = 161.85 on 4 degrees of freedom, *p* < 0.0001). Similar results were obtained when using mean ISR instead of median ISR. The outlier had no effect on these results. **B**. Trends in ISR values between 1990 and 2014 in the five wealth classes. Each thin line is a single country; the thick lines were fitted by local regression.

In developing countries, ISR is of course higher in richer segments of the population: averaged over all available data, ISR increases as we move from the poorest to the richest segments of developing-country populations (Fig 4A). ISR has also increased over time in all wealth classes (Fig 4B). However, it has increased faster in the poorer than in the richer fractions of the population. The estimated rates of increase per year for the poorest 20%, and the poorest 40% were, respectively, greater than the rates in the richest 20% and the richest 40% (the respective differences between rates being 0.10 (± 0.02 SE) and 0.07 (±0.02 SE)). These differences are statistically different (both p<0.0001 obtained by a linear mixed effects model including random intercepts for 985 observations of 72 countries).

Initial (1950-55) ISRs were on average lower in MACs (median, 82%) than in ODCs (median, 85%) and remained so through 2010-15: the highest observed ISR in a MAC is 96.3% (median, 94%) and in ODCs 99.8% (median, 98%) (Fig 2B).

### Fertility decline vs improvement in wellbeing in MACs and ODCs

UN data show that general wellbeing (as measured by ISR) improved later and more slowly in MACs than in ODCs. Our claim that this caused the later and slower fertility decline in MACs implies that improvements in wellbeing should be associated with similar declines in fertility in the two groups. We use two approaches to examine this.

*Between-group analyses*: We first compare TFR vs ISR in MACs and ODCs, as a whole, between 1950-55 and 2010-15.

**Fig 5:**
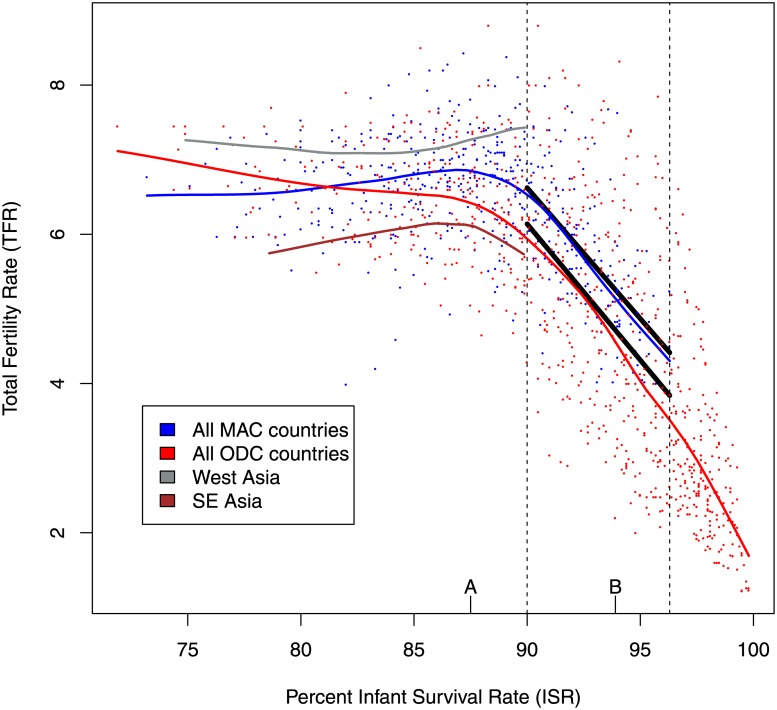
Each point is the TFR of a country during a 5-year period, plotted as a function of the country’s ISR in that period. Curves are local regression (LOESS) fits, using span = 1/3 [16]. Black regression lines are fitted in the approximately linear decline phase using all points with 90.0%≤ISR≤96.3%, indicated by vertical dotted lines. These have slopes -0.365 (ODC, 328 points, SE = 0.041) and -0.351 (MAC, 166 points, SE = 0.036), which are not statistically different. (In the linear model TFR = Mean + Region + ISR + Interaction, with independent, homoscedastic, Normal errors, the test for “no Interaction” gives *t* = 0.21, *p* = 0.84. When ODC and MAC error variances are not assumed equal, the slope estimates can be compared by a Welch t-test, which gives *t* = 0.26 and *p* = 0.79 on 380 degrees of freedom.) The extreme UN regions in the flat region (ISR ≤ 90%) are indicated by the olive and brown LOESS fits to West Asia and S.E. Asia, respectively. MAC and ODC slopes for 71.5%≤ISR≤87.5% are nearly flat (MAC slope = 0.04; ODC slope = -0.036) but differ statistically (the test for “no Interaction” gives *t* = 3.63, *p* = 0.0003). Markers A at 87.5% and B at 93.9% indicate the lower and upper limits for the range of slope overlap used in the single-country slope analyses (see S3 Text for further details).

Fertility declined approximately linearly, and at virtually identical speeds, in the shared range 90% ≤ ISR ≤ 96.3%. Linear regressions (black lines) fitted to the two groups over this range have statistically indistinguishable slopes (Fig 5). Reinforcing a conclusion of [7], MAC TFR was approximately half-a-child higher than ODC TFR at the start of the decline (see also Fig 2); because the two fertilities declined at the same speed, the half-child difference persisted. The speed of fertility decline in ODCs increased as ISR approached 100% (Fig 5).

Fertility decline began at approximately the same ISR values in MACs and ODCs (Fig 5). Median ISR in the year in which onset occurred (at a TFR 10% lower than the preceding highest value) was 91.18% in MACs and 92.05% in ODCs (MACs range of observations: 84.59%-95.29%; OCD range of observations: 83.46%-97.14%).

MAC and ODC trajectories are significantly different in the pre-decline phase (ISR ≤ 87.5%, point A in Fig 5), but the MAC trajectory was well within the large regional variation in ODCs seen in this phase. The slight decrease in ODC overall-group fertility in this phase was caused by differences among ODCs in initial (1950-1955) TFR values, not by fertility declines in individual countries: in the 34 ODCs that had initial ISR ≤ 85%, mean TFR change in this phase was -0.01 (± 0.71 SE).

*Single-country analyses*: Second, we measure change in fertility in individual countries over an ISR range that most countries in the two groups have experienced, and which also includes the ISR values where most fertility declines began. The range of overlap is 87.5% to 93.9% ISR (points A and B in Fig 5). Thirty-six MACs and 46 ODCs, have experienced ISR as low as 87.5% since 1950-55, and 90 of the 98 countries began their fertility declines at or above this threshold. The upper limit, 93.9%, is the median of the highest ISRs observed in MACs by 2010-15 and has been experienced by all but three ODCs.

**Fig 6:**
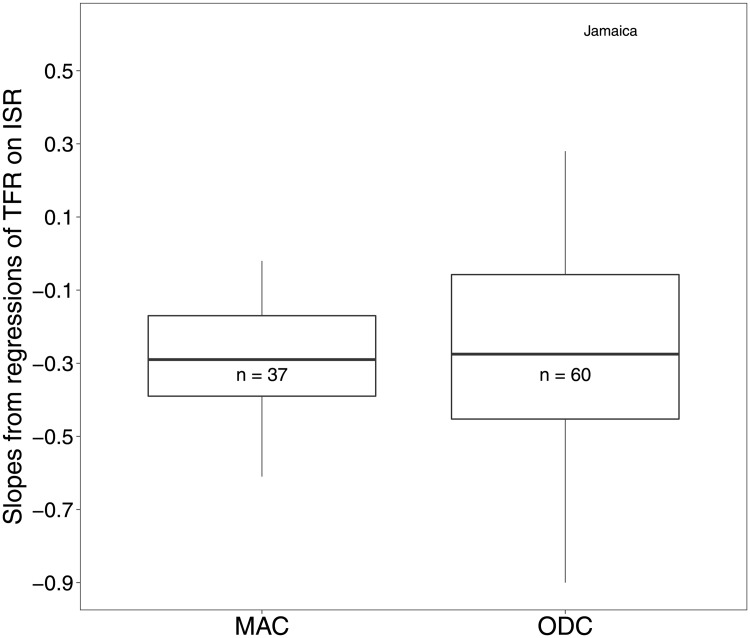
Results of regressions of TFR on ISR within the range 87.5% ≤ ISR ≤ 93.9%, for 60 ODCs, and in the range ISR ≥ 87.5% for 37 MACs. The black bars in the boxplots give the median slopes. The steepest regression slope for a MAC was -0.62, and for an ODC was -0.91. Mean slopes (± SE), MACs = -0.30 (± 0.15) and ODCs = -0.27 (± 0.28), are not significantly different (two sample t-test with unequal variance: *t* = −0.76 on 93.93 degrees of freedom, *p* = 0.45, sample size for ODC = 60 and MAC = 37). The outlier slope for ODC countries is Jamaica. Data from [3].

The speeds of decline of TFR vs ISR over this ISR range are statistically indistinguishable in the two groups (p>0.42 with two sample t-test for unequal variance, sample size for ODC = 60 and MAC = 37), though on average are slightly faster in MACs (Fig 6). The ODC group, which is geographically and culturally more diverse, shows a wider range of slopes.

### Population projections

We forecast population growth in the MACs by replacing what might be called the current “business as usual” scenario, which gives us the 11 billion projection for 2100, with scenarios where MACs achieve the faster improvements in wellbeing (indicated by ISR) that have been seen and projected in the ODCs, and hence faster declines in fertility. By contrasting these two scenarios we estimate the potential reduction in world population that can be achieved by actively investing in such accelerated improvement in wellbeing in the MACs.

The modeling machinery was that developed by Raftery and colleagues and now used by the UN [1, 17–19]. Population projections for each future 5-year interval for each country are based on many sequences of age-specific birth and age- and sex-specific death rates generated from probability distributions of these vital rate parameters, following procedures in BayesPopURL [20]. We used 1000 such sequences. Before replacing MAC rates by ODC rates in these calculations (as described below), we confirmed that using the MAC rates allowed us to replicate UN results: our median projected 2100 total MAC population (3.94 billion) was within 1% of the UN projection (3.97 billion). (See S4 Text for details.)

We then projected MAC populations based on historic and projected rates for 56 of the ODCs. (Following [1] we excluded from the ODCs the five nations affected by AIDS epidemics.) We first aligned the current (2010-2015) ISR of a given MAC with the matching ISR of each of the 56 ODCs (37 MACs x 56 ODCs = 2,072 MAC-ODC combinations). For example, Angola’s current ISR equals that of Laos in 1990-95. We then kept the first (2015-2020) set of UN-projected MAC rates but replaced the rest by the historic and projected ODC rates, beginning with the matching period. This delay assumes conservatively that even if wellbeing improves immediately, there is a lag of about 5 years before vital rates are affected. For example, we kept Angola’s 1000 sets of 2015-2020 rates (which affect its 2020-2025 population), replaced each set of its projected rates for 2020-2025 to 2040-2045 by Laos’s historic rates for 1990-1995 to 2010-2015, and replaced Angola’s sets of projected rates for 2045-2050 to 2095-2100 by Laos’s sets of projected rates for 2015-2020 to 2065-2070. This pattern—MAC rates for 2015-2020, then historic ODC rates, then projected ODC rates—holds in most cases.

In general, the initially-matched ODC and MAC ISR values will not be equal, so we find the two consecutive ODC periods that straddle the current MAC value, and choose the earlier (lower ISR value) period.

In 2% of 2,072 MAC-ODC combinations even the 1950-55 ODC ISRs are higher than the 2010-15 MAC ISR, and in another 2% the current ODC ISRs are lower than the current MAC ISR. We expand the matching criterion to treat these conservatively (see S4 Text).

**Fig 7:**
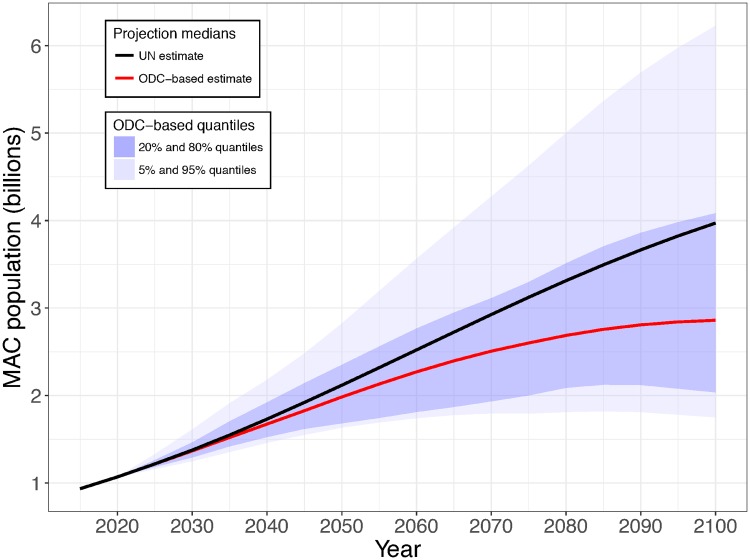
UN and ODC-based projected total MAC population (in billions). The intervals show quantiles of the distribution of our MAC population projections. More than 90% of their spread is due to variation in the historic and projected demography among the ODCs. The rest is due to the UN’s probabilistic trajectories. The calculations are described in S4 Text (Method B, median-adjusted). For a given MAC population projection, a value for the world population can be obtained by adding the total of the median UN projections for all other countries (7.24 billion).

Our projected ODC-based median total MAC population in 2100 is 2.86 billion (2.86B), which is 1.1B (28%) lower than the UN projection (Fig 7). Under these circumstances, world population in 2100 would be 10.1B, and stabilized by 2085. Improving wellbeing quickly is crucial: if MACs were to move earlier to ODC-based trajectories, in 2015-20, the 2100 projected median MAC total would be about 1.5 billion lower than the UN projection.

### Improving wellbeing in MACs

The results suggest that MAC fertility would fall as fast as it has done in the ODCs if wellbeing, exemplified by infant survival rate, were to increase at the speed it has done in the ODCs in similar demographic circumstances. This seems quite feasible. MACs are mostly poor, inequitable, corrupt and undemocratic; but most ODCs experienced similar conditions during their demographic transition.

Unusually rapid increases in ISR have been achieved at very low incomes and high levels of corruption. For the ODC countries in Fig 5, we recorded real per capita incomes [21] in the 5-year period when each first reached ISR ∼ 90%; the median income was $2387 (see S5 Text for details). We then calculated how rapidly (number of percentage points gained per 5-year period) ISR increased to 96% [3]. Eleven ODCs had increase rates ≥ 1.65%; three of them (Bangladesh, South Korea and Nepal) had among the lowest recorded incomes ($997-$1300) and S. Korea ($1300) had the highest recorded increase rate (2.6%). A fourth, Egypt, had the lowest income and the highest increase rate ($1740, 2.33%) among the five Muslim North African countries. (The other seven ODCs with rapidly increasing ISRs, mainly oil producers and all Muslim, were among the richest; they achieved 90% ISR very late, i.e. at high incomes.)

A focus on the poor has facilitated rapid increases in ISR at low per capita incomes. S. Korea (*c*.1960) was one of Asia’s poorest countries but had a famously equitable income distribution and land reform [22, 23]. The Bangladesh government explicitly expanded benefits (e.g. micro-credit to women, health care, increased free female schooling, access to contraception services) to poor and, especially, rural portions of the country, also using help from NGOs [24, 25]. It has achieved key 2015 UN Millennium Development Goals (MDGs), such as reducing maternal and childhood mortality and the fraction of the population in poverty, at extremely low average income levels. Controlled trials in Matlab, Bangladesh, showed reductions in childhood and maternal mortality and fertility in villages receiving outreach health and family planning services [25]. Nepal’s government likewise has committed to, and largely achieved, MDG targets. Among MACs, Rwanda, with ISR increase rate 2.0% and income only $1025 at ISR = 90%, exemplifies the effect of explicit government focus on achieving 2015 MDGs in a poor MAC [23].

Most encouragingly, MACs in general have achieved ISR ∼ 90% at much lower incomes (median = $1,283) than did the ODCs (median = $2,387). Furthermore, the nine MACs with ISR increase rates > 1.5% (1.57% to 2.25%) all had incomes ($344-$1171) below the MAC median.

With regard to corruption, in 2015 22 MACs ranked below (i.e. were worse than) 100 out of 167 countries, but 26 ODCs were also in this range [26]. Indeed, the rest of these 100 low-ranked countries all have ISR ≥ 98% except for three nations with population < 1M [26]. Bangladesh and Nepal have dismal histories of corruption. Bangladesh ranked last or second-to-last in the first four years of data (2002-2005), and by 2016 these two nations had escaped the bottom third in only two years [26].

**Fig 8:**
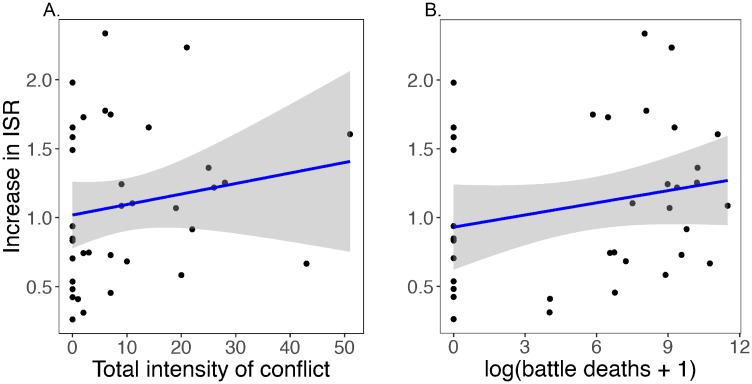
**A**. Speed of increase in ISR, between 1990-95 and 2010-15, versus overall intensity of civil conflict between 1990 and 2014 in 36 MACs (no data for S. Sudan). Each dot is a MAC. The slope of the relationship is not significantly different from 0 (slope = 0.008, *t* = 1.02 on 34 degrees of freedom, *p* = 0.32). **B**. Speed of increase in ISR vs log(cumulative number of battle deaths+1) in these countries. The slope of this relationship is not significantly different from 0 (slope = 0.03, *t* = 1.35 on 34 degrees of freedom, *p* = 0.19). Change in Rwanda, the point with coordinates (9.1, 2.2), is between 1995-2015 because the 1994 genocide temporarily and severely suppressed ISR to 71.9% in 1990-95. Shown are 95% confidence bands. Data from [27]. See S6 Text for more details on this figure.

Perhaps most surprising, in MACs since 1990 there is no correlation between the speed at which ISR has increased and the standard measures of civil conflict: frequency, intensity, and total deaths (Fig 8). Nepal again, is illustrative of this general point. Nepal achieved its relatively rapid increase in ISR even though its Maoist rebellion ended only in 2006, and it experienced almost 10,000 battle deaths between 1996 and 2006, close to the average number in MACs (10,127 deaths).

## Discussion

The UN’s median estimated world population for 2015 is 7.35 billion with 934 million people in the MAC countries. For 2100, its median projected populations are 11.2 billion and 3.97 billion respectively. Our ODC-based median projection for the MACs is 2.86 billion. Using the UN projections for all other countries, our median projected world population is 10.1 billion. Other sets of assumptions also lead to projections lower than the UN’s.

[28] made alternative population projections for sub-Saharan countries to determine the expected effect on future population size if the observed initial slow fertility decline were to be followed by an accelerated decline (then steady tapering) as seen in some other developing countries. For the projected sub-Saharan fertility patterns, [28] substituted observed fertility patterns seen in 21 other developing countries covering a range of social and economic contexts (e.g. Bangladesh, China, Peru). The resulting median projected 2100 sub-Saharan population was 770 million below the UN median projection, again emphasizing that significant slowing of growth is consistent with some historical experiences. Using the UN projections for all other countries, the projected world population is 10.4 billion.

[29] assumed universal implementation of several key UN (2015) Sustainable Development Goals by the target date of 2030: education through secondary school, specific reductions in maternal and infant mortality, and improved reproductive health and family planning. In their highly detailed model, education is the main driver of fertility and also increases survival rates and access to and use of family planning. Achieving these goals results in a boost to development and the demographic transition between 2015 and 2030; thereafter development and demographic changes occur at a more regular speed. Their model applies to all regions of the world, not just MACs or sub-Saharan Africa, and projects a total world population in 2100 of 8.19—8.65 billion.

Our projection is more empirical than that of [29]: we project on the basis of past experience, that of the ODCs. This experience varied widely, so our ODC-based projections do too: our 5% and 95% quantiles for the 2100 MAC population are 1.75 and 6.23 billion. If the MACs experienced fertility and mortality rate changes following those of S. Korea (the ODC with the fastest rate of increase in infant survival) or of Thailand, the median projected MAC population in 2100 would be only 1.75 billion. Using the UN projections for all other countries, our median projected world population would then be 8.99 billion. Basing future MAC rates on those of Bangladesh, Azerbaijan, Brazil or Myanmar would also lead to world projections which are at least 2 billion below the UN’s median of 11.2 billion.

These special cases are realistic. There is reason to hope that MAC infant survival could increase faster, and hence fertility decrease faster, than predicted by the overall ODC experience. As noted above, ISR reached 90% at a median income of $2,387 in ODCs, but of only $1,283 in MACs. Thus, while the projections of [29] are optimistic, the necessary demographic changes have been achieved in the past.

Our results and these others suggest that improving widespread wellbeing in MACs, at rates previously achieved by many other developing countries, is likely to lead to future populations much smaller than currently projected. A key step is to intensify current efforts to improve conditions in poor and rural areas, by governments (of MACs and more developed countries), international agencies and NGOs [22, 23].

## Supporting information

S1 Text Details relating to Fig 1: Country groups. (PDF)

S2 Text Fertility decline vs improvement in wellbeing in MACs and ODCs: other possible measures. (PDF)

S3 Text Fertility decline vs Infant Mortality Rate. (PDF)

S4 Text Population projection methods. (PDF)

S5 Text Data on per-capita income. (PDF)

S6 Text Data on armed conflicts. (PDF)
