# Supplementary material for: Improving wellbeing and reducing future world population
Source: PLoS One. 2018 Sep 12;13(9):e0202851. doi: 10.1371/journal.pone.0202851 (PMC6135380; doi:10.1371/journal.pone.0202851)
Supplement: S1 Text — (PDF) [file pone.0202851.s001.pdf]

S1 Text: Details relating to Figure 1: Country groups

We analyzed larger, demographically important nations by excluding countries with populations < 1 million in 2000; these are mainly small islands but, in the MACs, include two sub-Saharan mainland countries (Djibouti and Equatorial Guinea) and one northern African country (Western Sahara). The MACs also excluded the five, southern sub- Saharan African countries, which have already reached a TFR of 2.51.

We used one of two criteria to determine the ODC group. (1) The country had TFR ≥ 6 in 1950-55 (the first period for which UN world data are available) or later. (2) If TFR never reached 6, it reached and exceeded 5.5 and increased or stayed level for at least two estimation periods before decreasing. For example, India's first three estimated values were: 5.9, 5.9 and 5.89; Jamaica's first four were: 4.22, 5.08, 5.64, and 5.78. The TFRs of nine of the 61 ODCs never reached 6. Of the 37 MACs only two had maximum TFR < 6: Central African Republic (max TFR = 5.95) and Gabon (max TFR = 5.72). Low initial fertilities in MACs and some ODCs in South Asia were likely induced by disease-related infertility [1–3], but a few ODCs may have begun fertility decline before data became available in 1950-55; however, they passed through the great majority of their declines thereafter. We excluded China because of its unique 1-child policy.

References

[1] Frank O. Infertility in sub-Saharan Africa: estimates and implications. Population and Development Review. 1983;9(1):137–144.

[2] Mascarenhas MN, Flaxman SR, Boerma T, Vanderpoel S, Stevens GA. National, regional, and global trends in infertility prevalence since 1990: A systematic analysis of 277 health surveys. PLoS Medicine. 2012;9(12):1–12. doi:10.1371/journal.pmed.1001356.

[3] Williams J, Ibisomi L, Sartorius B, Kahn K, Collinson M, Tollman S, et al. Convergence in fertility of South Africans and Mozambicans in rural South Africa, 1993-2009. Global Health Action. 2013;6:19236. doi:10.3402/gha.v6i0.19236.
