# Supplementary material for: Improving wellbeing and reducing future world population
Source: PLoS One. 2018 Sep 12;13(9):e0202851. doi: 10.1371/journal.pone.0202851 (PMC6135380; doi:10.1371/journal.pone.0202851)
Supplement: S2 Text — (PDF) [file pone.0202851.s002.pdf]

S2 Text: Fertility decline vs improvement in wellbeing in MACs and ODCs: other possible measures

Life Expectancy (LE) is a possible alternative proxy for overall health and wellbeing, and is a component of the UN Human Development Index. ISR has the advantage of being a more immediate reflection of health status whereas life expectancy (LE), with which it is highly correlated, reflects both current and past health conditions. The correlation between ISR and LE over 1950 to 2015 for all 98 countries in this study is 0.93 (Pearson correlation measure). In addition, Angeles (2010) investigated lagged relationships in 118 developed and developing nations between 1960 and 2000. He found that, while both measures predict TFR better if they are lagged, the estimated average lag is shorter for ISR (10 years) than for LE (20 years).

Educational level is also a possible proxy for wellbeing. Data on years of schooling are available for 1950 onwards [1]. Unfortunately, data are not available for 30% of MACs and 10% of ODCs. A second problem is that years of schooling may imply different levels of learning and skills in different countries and regions. [2] show, for example, that a 9th grader in Honduras is more than 6 years behind an average 9th grader in Singapore in terms of cognitive skills as measured by standard tests. They suggest this difference reflects not only the schools themselves, but also the cultural milieu. Cognitive skills are only one aspect of the benefits of schooling, but other benefits are also likely to vary among countries. By contrast, the number of infant deaths per 1000 live births is an unambiguous variable. [3] provide evidence that mother's education affects infant mortality.

References

[1] Barro RJ, Lee J. Barro-Lee Education Attainment Dataset; 2013. Available from: `http://www.barrolee.com/`.

[2] Hanushek EA, Woessmann L. Knowledge capital, growth, and the East Asian miracle. Science. 2016;351(6271):344–345. doi:10.1126/science.aad7796.

[3] Fuchs R. Education or wealth: Which matters most for reducting child mortality in developing countries. Vienna Yearbook of Population Research. 2010;8:175–199.
