# Supplementary material for: Improving wellbeing and reducing future world population
Source: PLoS One. 2018 Sep 12;13(9):e0202851. doi: 10.1371/journal.pone.0202851 (PMC6135380; doi:10.1371/journal.pone.0202851)
Supplement: S3 Text — (PDF) [file pone.0202851.s003.pdf]

S3 Text: Fertility decline vs Infant Mortality Rate

Between-group analyses: Data from Rwanda (1990-95) and Cambodia (1975-80) are omitted from Fig. 5 because genocide and massacres temporarily increased infant mortality rates to extremely high values. In both cases, there was a surprisingly rapid return to a decreasing trend. In Rwanda, the sequence of ISRs in the four 5-year periods beginning in 1985-90 was 88.6%, 71.1%, 88.4% and 91%. In Cambodia the sequence, starting in 1970-75, was 86.1%, 68%, 84.6% and 91.4%.

Single-country analyses: Because initial ISR values were lower and ISR increased more slowly in MACs than in ODCs, MACs had more data points in general in the single-country regressions (median = 8, range = 2-13; 1 country had 2 observations) than did ODCs (median = 4, range = 2-9; 7 countries had 2 observations).

The great majority of countries (77 of 89) with more than 2 observations yielded linear regressions with $R^2 > 0.5$; 57 had $R^2 > 0.75$. Among low correlations ($R^2 < 0.5$) there is either almost no change in TFR plus only slight deviation from linearity (5 countries) or there is strong non-linearity because, as ISR increases, TFR first increases then declines (5 countries). Two nations (Congo and Zimbabwe) show strong nonlinearity during the decline phase, which may be related to the prevalence of AIDS or other traumas.

References
