# Supplementary material for: Improving wellbeing and reducing future world population
Source: PLoS One. 2018 Sep 12;13(9):e0202851. doi: 10.1371/journal.pone.0202851 (PMC6135380; doi:10.1371/journal.pone.0202851)
Supplement: S4 Text — (PDF) [file pone.0202851.s004.pdf]

S4 Text: Population projection methods

We project MAC populations through 2100 based on the approach developed by Raftery and colleagues [1–4], and now used by the UN.

UN Methods

The UN's population projections use forecasts of TFR and male and female life expectancy (e0M and e0F) over time for 201 countries and 32 other areas. The forecast formulae contain country-specific parameters and perturbations. Both contribute randomness to the forecasts.

In what follows, we refer to predictions of future TFR or e0 as "forecasts" and predictions of future populations as "projections." Each consists of 17 values: every 5-year period from 2015-20 to 2095-2100 for forecasts, and every 5 years from 2020 to 2100 for projections.

The parameters and perturbations are modeled as if they arose in two steps. Each involves "prior" distributions reflecting weak assumptions that make no use (or minimal qualitative use) of the UN data. First, "world" parameters were randomly chosen from "known" prior distributions (i.e., specified by the modeler). Some world parameters defined distributions from which the perturbations were chosen; others were used in the second step, to define further prior distributions from which each country's specific parameters were chosen.

These parameters apply to past as well as future values. Thus, the UN's TFR and e0 data from all countries, for the periods 1950-55 to 2010-15, can be used to update the prior distributions for the world parameters, giving conditional distributions. These lead to new priors for the country-specific parameters, which can be updated to obtain conditional distributions for each country, using only its own data. Each country has the same new prior distributions (since the world parameters apply to all), but they have different conditional distributions because of different past data.

To make a probabilistic forecast, we first choose world and country-specific parameters in the same two steps, but using the conditional distributions. The resulting set of parameters is a "trajectory." The parameters (and the random perturbations) then give the TFR and e0 forecasts for each country. We can repeat this process (choose trajectory, calculate forecasts) as often as we like.

Our MAC forecasts and projections

The BayesPop website [5] gives 1000 trajectories. We had to alter their forecasts slightly. The UN changed the website's prior distributions for some country-specific e0F parameters because they led to implausible forecasts; for example, rapid recent improvement (perhaps due to lower child mortality) caused some countries with currently low life expectancies being forecast to catch and surpass others with higher ones (see pg. 26-27 of [6]). We used the forecasts from the website's trajectories but, for each country and year, added a constant to our 1000 e0F values so their median equaled the median of the corresponding UN values. We did this for e0M and TFR as well.

A trajectory's forecasts are converted to age-specific rates of fertility and mortality, as described by [5]. Beginning with each country's 2015 population, these rates are used to project future populations by the cohort-component method. Thus, each trajectory yields a projection for each country. The sum of these projections over the countries in a region is the trajectory's regional projection.

With 1000 trajectories, each country has 1000 projections; its median projection is the median of these; other quantiles are similar. Medians for regions can be defined the same way, but an alternative method is described under "Comparing Projections" below.

Our ODC-based forecasts and projections

Our hypothesis is that if well-being (ISR) improves in MACs as rapidly as seen or expected in ODCs, future MAC populations will be lower than currently projected. For each (MAC, ODC) pair, we assume the MAC's future ISR improves at the same rate as the ODC's, and that this leads to the future sequences of TFR, e0F and e0M for the MAC being the same as were seen or forecast for the ODC. We substitute these ODC sequences for those forecast for the MAC, and compare the resulting population projections with the original MAC projections.

We first describe our substitution rules. In our description, we use mid-years (e.g., 1953, 2098) to represent periods (1950-55, 2095-2100). Values of ISR, e0 and TFR for years 1953-2013 are observed; the rest are forecasts. However, for brevity, we call them all "forecasts."

We find an ODC year, YO $>$ 1953, which "matches" a MAC year (YM $>$ 2013, ideally 2013) in that the ODC's ISR in YO is close to the MAC's in YM. We could replace the MAC's TFR and e0 forecasts for years YM + 5, YM + 10, etc., by the ODC forecasts for years YO + 5, YO + 10, etc., but we introduce a delay to be conservative: we keep the MAC's YM + 5 values, and replace its values for YM + 10, YM + 15, ... by the ODC's values for YO + 5, YO + 10, ...

We also require the ODC's ISR in YO to be less than the MAC's in YM.

Thus, all ODC-based MAC projections use the MAC's own 2018 forecasts of TFR and e0 to project the 2020 population. If YM = 2013, the MAC's 2023 forecasts are replaced by the ODC forecasts for YO + 5 to project the 2025 population. If YM > 2013, an ODC ISR is matched with a forecast MAC ISR; then the MAC's own forecasts are used for years 2018, 2023, . . . , YM + 5, and the ODC's forecasts for YO + 5, ..., are used after that.

Formally, for each ODC, we made an ISR vector of 30 components: IO[Y] = ODC's ISR value in year Y, for Y = 1953, . . . , 2013, 2018, . . . , 2098.

For each MAC, we made an ISR vector of 18 components: IM[Y] = MAC's ISR value in year Y, for Y = 2013, 2018, . . . , 2098.

Now define YO to be the first year in which IO[YO] < IM[YM] <= IO[YO + 5] for some year, YM, in the MAC vector. Then the ODC-based forecast of (say) TFR for this MAC would be:

i. For years 2013, 2018, ..., YM + 5, the MAC values;

ii. For years YM + 10, . . . , 2098, the ODC values for years YO + 5, ... YO + K,

where K = 2093 - YM.

For the 2072 (MAC, ODC) pairs, this procedure gives YM = 2013 in 1,985 cases. For example, Angola's 2013 ISR is 90.4%; Iran's values for 1973 and 1978 are 87.6 and 90.5; thus YM = 2013 and YO = 1973, since IO[1973] (= 87.6) < IM[2013] (= 90.4) ≤ IO[1978] (= 90.5). Thus, the Iran-based projection for Angola uses Angola's TFR and e0 forecasts for 2018, but Iran's 1978 forecasts for 2023.

There are 43 cases with IO[1953] > IM[2013], so the MAC's 2013 ISR is less than all the ODC's values. For example, Chad's values for 2013, 2018 and 2023 are 90.4, 91.3 and 92.2; Panama's 1953 and 1958 values are 91.4 and 92.6. Thus YM = 2023 and YO = 1953, since IO[1953] < IM[2023] ≤ IO[1958]. The Panama-based projection for Chad uses Chad 's TFR and e0 forecasts up to 2028, but Panama's 1958 forecasts for 2033.

There are 19 cases with IO[2013] < IM[2013] but the forecast ISR increases sufficiently faster for the ODC than for the MAC that the criterion can be satisfied. For example, Pakistan's 2013 ISR is 93, while Gabon's is 95.7; but Pakistan's values for 2073 and 2078 are 98.2 and 98.5, and Gabon's for 2068 is 98.4, so YM = 2068 and YO = 2073. The Pakistan-based projection for Gabon uses Gabon's TFR and e0 forecasts up to 2073, but Pakistan's 2078 forecasts for 2078.

Finally, there are 25 cases with IO[2013] < IM[2013] where the criterion gives YM > 2088,

so there are no years in the sequence YO + 5, ... YO + K above. For these (MAC, ODC) pairs, the "ODC-based" projection of the MAC population was the MAC's own original projection.

There were 37 MACs, 56 ODCs and 1000 trajectories. For 24 MACs, this procedure gave a population projection for each combination of an ODC and a trajectory. Due to these last 25 cases, eight MACS could be combined with only 55 ODCs, three with 54, one with 53 and one (Madagascar) with 48.

Comparing UN and ODC-based MAC Projections

Some choices need to be made in calculating projected regional (e.g. total MAC) median and quantiles of the population. For a single country, a projected value is produced for each trajectory (see above). To project the sum of all the MAC populations, we can sum the country projections for each trajectory and then obtain quantiles over the trajectories. Trajectories are independent, but projections for different countries based on the same trajectory are correlated since the common world parameters affect both perturbations and country-specific parameters. The correlation increases the spread of the sum, but some correlation is likely since the MACs are near each other [7].

The UN adjusts this "trajectory-based" regional method. Its "median" projection for the region is defined as the sum of the median projections for the countries in it. This is usually different from the median of the trajectory-based sums. Thus, a constant (UN median - trajectory-based median), is added to each trajectory-based quantile, to obtain a set of adjusted quantiles. The adjustment is usually small. We follow these procedures.

This does not introduce a "bias." The median of a sample is not usually an unbiased estimate of the median of the underlying distribution. The aim here is to describe the center and spread of the projections with only minor distortion due to extreme observations. If each country's set of projections is positively skewed, then occasional very large projections could affect the trajectory-based median of the sum but will not affect the UN median. The UN median is also natural in the sense that a reader might expect country estimates to add to the regional estimate.

For a given MAC, options for the "median" ODC-based projection include:

1. find the median of all projections (56 ODCs $\times$ 1000 trajectories);
2. find the median over trajectories for each ODC; get the median of these medians;
3. find the median over ODCs for each trajectory; get the median of these medians.

The answers differ, though by far less than each does from the UN median or our MAC-based median. Only (1) extends directly to other quantiles.

We marginally prefer to use median (2) as the "center" estimate, and to adjust the quantiles obtained from (1) by adding median (2) - median (1) to each. This seems closer to the UN approach when distributions are to be combined, and emphasizes the variation over the non-random ODCs rather than the trajectories.

For quantiles of projections of sums of the MACs we considered three options:

A. For each ODC and trajectory, sum the projections over MACs. Get quantiles of these 56,000 sums.

B. For each MAC and ODC, get the median over trajectories. For each ODC, add these medians over MACs. Get quantiles of these 56 sums.

C. For each MAC and trajectory, get the median over ODCs. For each trajectory, add these medians over MACs. Get quantiles of these 1,000 sums.

We prefer method (B), for reasons like those before. All three methods can be adjusted so that the center is the sum of the MAC medians, as given by (1), (2) or (3). We prefer to adjust by (2), so our quantiles are given by quantiles(B) + (Sum of medians (2) - median(B)).

With our 5-year delay (so most ODC-based rates begin in 2023 rather than 2018), our median ODC-based projection for the sum of the MACs in 2100 is 2.86 billion. Without the delay, it is 2.43 billion. The UN's median projection is 3.97 billion.

MAC projections do not include international migration, which would reduce our projected total MAC population for 2100 by approximately 50 million [7]. Moreover, out-migration from MACs, inevitably to lower-fertility countries, may have negligible effects on fertility in non-MACs as there is evidence that the migrants' fertility declines to levels prevailing in the recipient nation [8].

References

[1] Alkema L, Raftery AE, Gerland P, Clark SJ, Pelletier F, Buettner T, et al. Probabilistic projections of the total fertility rate for all countries. Demography. 2011;48(3):815–839. doi:10.1007/s13524-011-0040-5.

[2] Raftery AE, Li N, Ševčíková H, Gerland P, Heilig GK. Bayesian probabilistic population projections for all countries. Proceedings of the National Academy of Sciences. 2012;109(35):13915–13921. doi:10.1073/pnas.1211452109.

[3] Raftery AE, Alkema L, Gerland P. Bayesian population projections for the United Nations. Statistical Science. 2014;29:58–68.

[4] Gerland P, Raftery AE, Ševčíková H, Li N, Gu D, Spoorenberg T, et al. World population stabilization unlikely this century. Science. 2014;346(6206):234–237. doi:10.1126/science.1257469.

[5] Ševčíková H, Raftery AE. BayesPop: Probabilistic population projections. Journal of Statistical Software. 2016;75(5):1–29.

[6] Garenne M. Education and fertility in sub-Saharan Africa: A longitudinal perspective. Calverton, Maryland, USA: ICF International; 2012.

[7] United Nations. World population prospects: Methodology of the United Nations population estimates and projections. New York: United Nations; 2015. Available from: https://esa.un.org/unpd/wpp/.

[8] Williams J, Ibisomi L, Sartorius B, Kahn K, Collinson M, Tollman S, et al. Convergence in fertility of South Africans and Mozambicans in rural South Africa, 1993-2009. Global Health Action. 2013;6:19236. doi:10.3402/gha.v6i0.19236.
