# Supplementary material for: Improving wellbeing and reducing future world population
Source: PLoS One. 2018 Sep 12;13(9):e0202851. doi: 10.1371/journal.pone.0202851 (PMC6135380; doi:10.1371/journal.pone.0202851)
Supplement: S5 Text — (PDF) [file pone.0202851.s005.pdf]

S5 Text: Data on per-capita income

Per capita incomes were obtained by dividing the “Output-side real GDP at chained PPPs (in mil. 2011US$)” in the Penn World Table [1] by the concurrent population (in millions). The historic values are from the mid-point of the 5-year period for which the relevant ISR has been estimated. When estimates begin only after that year, the number is for the closest year up to five years later. No estimates are available within this time limit for a few countries (Kuwait, Libya, Turkmenistan, Uzbekistan, and Papua New Guinea). Among MACs, estimates are not available for Eritrea, Somalia and South Sudan.

References

[1] Feenstra RC, Inklaar R, Timmer MP. The next generation of the Penn World Table. American Economic Review. 2015;105(10):3150–3182.
