# Supplementary material for: Improving wellbeing and reducing future world population
Source: PLoS One. 2018 Sep 12;13(9):e0202851. doi: 10.1371/journal.pone.0202851 (PMC6135380; doi:10.1371/journal.pone.0202851)
Supplement: S6 Text — (PDF) [file pone.0202851.s006.pdf]

S6 Text: Data on armed conflicts

Data on armed conflict since 1946 have been collected and presented by the Uppsula Conflict Data Program at the Department of Peace and Conflict Research, Uppsula University [1]. We did two analyses for the period 1990-2014.

First, the database contains the number of battle-related deaths (soldiers and civilians killed in combat in state-based armed conflict) in each year in each nation, and for each nation we calculated the total number of deaths over the period 1990-2014. Second, we calculated the total intensity over the period. In the database, for each calendar year, conflict resulting in 25-999 battle-related deaths per year is scored an intensity of "1" (the most frequent score); a year with $\geq$ 1000 deaths is scored "2". For each country, we summed the scores over the period to obtain a measure that combines frequency and intensity of conflicts over the period.

References

[1] Peace Research Institute Oslo. Data on Armed Conflict; 2008. Available from: `https://www.prio.org/Data/Armed-Conflict/`.
